# Dental Education

**Published:** 1885-01

**Authors:** 


Dental Education.—After the present session of 1884-'85, five or even more years of dental practice will not be considered equivalent to one session in any of the reputable dental schools of this country. While we cheerfully acquiesce in the regulations adopted by the American Dental Association and the National Board of Dental Examiners, yet we hold to the opinion that five or more years of actual dental practice are, and should be considered a better qualificaiton for a dental student to present himself as a candidate for graduation, after attending one full session of five months in a dental school, than attendance on one course of medical lectures even wtih two years of dental office pupilage.

We also perceive that the Association of Dental Faculties has adopted a graded course for themselves, wherein the first course or junior students are confined to mechanical dentistry for the first session. and not permitted to begin the practice of operative dentistry until the second session. This, we think, is a decidedly backward movement, and one that will be productive of great injury to the cause of dental education. So far from being prevented from taking advantage of all the opportunities afforded by two full sessions in operative dentistry, we contend that the entire time of two sessions of five months each is absolutely necessary to qualify the dental student in this branch of his profession ; and that it wodld be much better to increase instead of to diminish the time for such study, and experience. Such a restricted course ofstudy will, perhaps, answer the purpose of enabling those schools that have a limited supply of clinical material, in the form of infirmary patients, to supply their senior classes to the exclusion of their junior classes and we are inclined to believe that such was the motive that instigated the adoption of such a graded course, or at least its presentation for adoption. It is certainly a great and deplorable mistake to deprive dental students of any advantages which dental schools can afford them, and such action must have the effect of lowering the standard of dental qualification.

